# An Expanded Access Program With NexoBrid for Treatment of Acute Deep Partial and Full Thickness Burn Injuries in Adult and Pediatric Patients

**DOI:** 10.1093/jbcr/irag075

**Published:** 2026-05-19

**Authors:** Steven A Kahn, John Schulz, Tam Pham, Andrew Clinton Bright, Dhaval Bhavsar, Nicole Bernal, Steve Sandoval, Robert Bertellotti

**Affiliations:** Burn Surgery, Medical University of South Carolina, Charleston SC 29425, United States; Division of Burns, Massachusetts General Hospital, Boston, MA 02114, United States; University of Washington Regional Burn Center, Seattle, WA 98104, United States; Department of Surgery, USA Health University Hospital, Mobile, AL 36617, United States; Department of Plastic Surgery, University of Kansas Medical Center, Kansas City, KS 66160, United States; Wexner Medical Center, The Ohio State University, Columbus, OH 43210, United States; Burn Unit, Renaissance School of Medicine, Stony Brook University, Stony Brook, NY 11794, United States; Acute Surgery, Carver College of Medicine, Iowa City, IA 52242, United States

**Keywords:** NEXT, NexoBrid, enzymatic debridement, modified Vancouver scar scale (MVSS), burn mass casualty incident (BMCI)

## Abstract

Following global Phase III trials, a single-arm expanded access program at 23 burn centers in the United States (2019-2024) provided centers with additional experience in treating adult and pediatric burn patients with NexoBrid, and maintaining burn care preparedness for mass casualty incidents. Eligible patients included children (<18-years-old) and adults (≥18-years-old) with deep thermal burns covering up to 30% of the total body surface area. NexoBrid application was followed by standard care. Patients were monitored weekly until wound closure, and again after 3 and 12 months. Outcomes included incidence and time to eschar removal, need for surgical excision or escharotomy, length of hospital stay, wound closure, and Modified Vancouver Scar Scale. A total of 239 patients (215 adult, 24 pediatric) received NexoBrid, with 142 (131 adult, 11 pediatric) completing the 12-month follow-up. Mean ages were 41 and 11 years, respectively. The mean treated target wound area was approximately 6% of the total body surface area, with 38 circumferential burns. Eschar removal was achieved in 95% of adults and 100% of pediatric patients within 4 hours. Surgical excision was performed in 4% of adults, but not in pediatric cases. No escharotomies were needed. The median length of stay was 10 days. Wound closure occurred by 22 days (adults) and 28 days (pediatric). Safety data were consistent with previous trials. NexoBrid demonstrated comparable outcomes vs previous Phase III trials and potential efficacy in preventing burn-induced compartment syndrome.

## INTRODUCTION

Burn injuries remain a significant global health issue, with the 2024 Annual Burn Injury Summary Report estimating that 1 in 10 000 individuals in the United States receive in-patient hospitalization in specialized burn centers annually. In 2023, more than 10 000 burn cases necessitated intensive care, collectively utilizing 110 000 intensive care unit days.^1^ Globally, despite a trend toward reduced burn-related mortality, the World Health Organization estimates that 180 000 deaths are caused by burns each year, predominantly in low- and middle-income countries.^2^ The short- and long-term management of burns can profoundly affect the functional abilities, comorbidities, and quality of life of survivors.

Eschar removal (burn excision) is the first and most critical stage in preventing eschar-related complications and initiating the healing process. Timely escharectomy reduces inflammation, infection, and burn propagation; prevents or resolves burn-induced compartment syndrome (BICS); enables accurate wound assessment and diagnosis; supports timely healing and improves scar outcomes.^3^ The current standard of care for eschar removal^4^^,^^5^ involves both non-surgical and surgical methods. The strategy for eschar removal is based on various factors such as burn depth, anatomical location, size (expressed as a percentage of total body surface area [TBSA] of the burn wound), the patient’s general condition, and the availability of donor sites for autograft harvesting. In addition, the availability of surgical facilities and personnel is an important factor, since surgical procedures require skilled personnel and sophisticated medical resources. The limitations of tangential excision in preserving viable tissue remain a concern. Dr. Zora Janzekovic emphasized the importance of preserving viable dermis through tangential excision, the meticulous, layer-by-layer removal of non-viable tissue until a viable wound bed is reached.^6^ This approach is particularly critical in pediatric patients, whose skin is significantly thinner and more vulnerable to excessive tissue loss. Thus, surgical eschar removal poses significant challenges.

NexoBrid is an alternative, non-surgical debridement modality. Composed of proteolytic enzymes enriched in bromelain, extracted from the stem of the pineapple plant (*Ananas comosus* [L.] Merr.), NexoBrid rapidly and selectively degrades and removes burn wound eschar and non-viable tissues, leaving a wound bed that can support spontaneous re-epithelialization. Bromelain-based debridement is performed at the bedside, without the need for specialized equipment. Three pivotal studies involving adult and pediatric populations demonstrated complete eschar removal in more than 90% of treated patients, with significantly reduced need for surgical excision, reduced time to complete escharectomy, decreased blood loss during excision, and a safety profile comparable to the standard of care.^7–9^ In addition, an important advantage of enzymatic debridement is its potential to reduce the exposure of pediatric burn patients to the physical and psychological trauma associated with major surgery and general anesthesia.^10^ A meta-analysis of publications from 2013 to 2023 regarding NexoBrid treatment revealed benefits including faster complete eschar removal and healing times, reduced operations, shorter hospital stays, fewer cases of sepsis, decreased blood transfusions, and prevention of compartment syndrome.^11^

NexoBrid was first approved by the European Medicines Agency in 2012 for eschar removal in adults with deep thermal burns,^12^ and in 2023, the indication was expanded to include the pediatric population. In 2022, it received approval from the US Food and Drug Administration (FDA) and the Japanese Pharmaceuticals and Medical Devices Agency (PMDA) for eschar removal in adult patients with deep partial thickness (DPT) and/or full thickness (FT) thermal burns. In 2024, this indication was expanded to include pediatric patients. As of 2025, NexoBrid is approved for use in 44 countries. These approvals were based on results from 3 large, controlled phase 3 trials: MW 2004-11-02 trial in adults and children,^13^ the DETECT trial in adults,^11^ and the CIDS trial in children,^10^ which concluded that NexoBrid resulted in complete and more rapid eschar removal, less blood loss, reduced the need for excisional surgery, and had a safety profile comparable to standard of care with no adverse effects on wound closure, cosmesis, or function.

Here, we present results from the NexoBrid Expanded access Treatment program (NEXT) in the United States, initiated to allow treatment with NexoBrid and enrich health care providers’ experience with its use for eschar removal in adult and pediatric burn patients, and conducted at US clinical centers that had previously participated in either the DETECT or CIDS clinical trials. An additional objective of the NEXT expanded access program (EAP) was to sustain readiness for burn care during mass casualty situations in the United States.

## MATERIALS AND METHODS

### Design and setting

This was a non-randomized, single-arm, open-label, EAP providing treatment with NexoBrid to 239 patients (215 adult, 24 pediatric) in 23 burn centers in the United States. The EAP was registered with ClinicalTrials.gov identifier NCT04040660.

### Study stages and inclusion criteria

The EAP included male and female adult and pediatric patients with thermal burns.

Enrollment in the EAP comprised 3 stages, pending recruitment status in DETECT and CIDS, and US approval status:

1. Stage 1—only adults (≥18 years of age).
2. Stage 2—adults and children (<18 years of age). Stage 2 was terminated following FDA approval of the product for the adult population.
3. Stage 3—only children.

Eligible patients had total target burn areas of which at least 1% consisted of DPT or FT burns requiring eschar removal according to the standard of care. The total burned area, as measured by %TBSA, could not exceed 30% (superficial partial thickness, DPT, or FT). The target wound (TW) was defined as a continuous burn area composed of DPT and/or FT burn areas that could be treated with NexoBrid. The treating physicians could identify one or more TWs per patient. TWs could be a portion of a larger wound that included face, genital, or perineal areas, although these areas were not included in the TW area. Circumferential wounds without prior escharotomy were also included.

Patients with electrical or chemical burns, wounds saturated with silver, iodine or by Silver Sulfadiazine (SSD), infections, pregnant individuals, those with poorly controlled diabetes mellitus, a body mass index of ≥40.0 kg/m^2^, severe cardiovascular or pulmonary diseases, or any conditions that would preclude safe treatment were excluded from the program.

Informed consent was obtained from each patient or parent/legal guardian (for under-age patients). The assent of pediatric patients was also obtained, where appropriate.

### Intervention

NexoBrid is supplied as a lyophilized powder and a gel vehicle for preparation of a gel for cutaneous use. Each gram of the prepared product contains 8.8% of proteolytic enzymes enriched in bromelain. The powder and gel components are mixed no more than 15 minutes prior to use to maximize enzyme activity at the time of application.

Prior to NexoBrid application, wounds were thoroughly cleansed with saline, soap, or an antibacterial solution. Superficial keratin layers, including blisters, were carefully removed, and the wounds were subsequently soaked in an antibacterial solution (eg, 0.25% sodium hypochlorite) for a minimum of 2 hours. This preparation ensured a clean, moist eschar-covered wound bed suitable for enzymatic debridement.

Appropriate prophylactic analgesia and/or sedation, as considered suitable for extensive dressing change, was administered prior to application. In adult patients, NexoBrid gel was topically applied to the burn wound at a dosage of 2 g NexoBrid sterile powder mixed with 20 g of sterile gel vehicle per 1% of TBSA (approximately equivalent to 180 cm^2^) or 5 g of NexoBrid sterile powder mixed with 50 g of sterile gel vehicle per 2.5% of TBSA. Pediatric patients received a similar dosage of 2 g of NexoBrid sterile powder mixed with 20 g of sterile gel vehicle per 180 cm^2^. Treatment with NexoBrid was administered within 84 hours of the burn injury. A Vaseline-based barrier was applied a few centimeters beyond the margins of the treatment area. The wound was then covered with an occlusive film dressing, which was secured by adherence to the surrounding barrier and reinforced with an overlying gauze dressing. NexoBrid was left in place on the burn wound for 4 hours, after which it was removed, along with the liquefied eschar. The wound was then soaked for 2 additional hours.

Patients with TWs of up to 15% TBSA were usually treated with a single NexoBrid application for 4 hours. Those with TWs exceeding 15% TBSA, up to a maximum of 30% TBSA, were treated with 2 consecutive applications, each lasting 4 hours. NexoBrid was not applied to more than 15% TBSA in a single session. Complete eschar removal was defined as the removal of ≥95% of the eschar.^5^ If less than 95% was removed following the first application, a second NexoBrid application could be performed no later than 24 hours after the initial application.

In patients with circumferential extremity wounds, pressure measurements were performed before treatment initiation, closely monitored throughout treatment, and repeated after NexoBrid removal. Specific circumferential extremity TWs were monitored for the following clinical signs of BICS: (1) increasing interstitial/compartment pressure > 30 mmHg; (2) difference of >20 mmHg of diastolic pressure between the injured and opposite uninjured limb; (3) decreased SpO_2_ reading during treatment with a difference of >6% compared to a non-injured extremity or deterioration of clinical status; and (4) deterioration of any of the 5 “P” signs: pain, paralysis, pulselessness, pallor, paresthesia.

Following eschar removal, patients underwent daily vital sign and pain assessments for 3 days from the morning following the start of eschar removal. Pain assessment was recorded using the Numeric Pain Rating Scale (NPRS), with scores ranging from 0 to 10, with 0 representing no pain and 10 the greatest pain possible, and the Face Pain Scale-Revised (FPS-R), with scores ranging from 0 to 5, with 0 representing no pain and 5 the greatest pain possible.

Blood tests (hematology, biochemistry, and coagulation) were performed 24 ± 6 hours post-eschar removal. Weekly assessment of wound healing progress, including the dressings used until complete wound closure, was performed. Assessment of scars by the Modified Vancouver Scar Scale (MVSS) was performed at 3 and 12 months (for evaluable patients) after the wound closure confirmation visit.

### Data collection and outcomes

Clinical performance evaluation included incidence of complete eschar removal, time to complete eschar removal, incidence and percent area excised during eschar removal procedure, incidence of surgical escharotomy procedures on circumferential TWs in extremities, and duration of hospitalization.

Safety data collected included adverse events (AEs), vital signs, laboratory tests, and pain assessments. In addition, blood transfusions given during hospitalization and time to complete wound closure (defined as re-epithelialization without drainage or dressing requirements confirmed at 2 consecutive visits, 2 weeks apart^14^) were recorded.

Cosmesis and function assessment were performed by a burn wound specialist based on the MVSS at 3 and 12 months (in patients who were followed for 12 months) after the wound closure confirmation visit.

### Statistical analyses

All statistical analyses were descriptive in nature; no pre-defined hypothesis testing was performed. Estimates of clinical performance and efficacy outcome parameters, including wound closure rate, median time to wound closure, and the average MVSS score, were presented with corresponding 95% confidence intervals.

## RESULTS

### NEXT population

**Figure 1:**
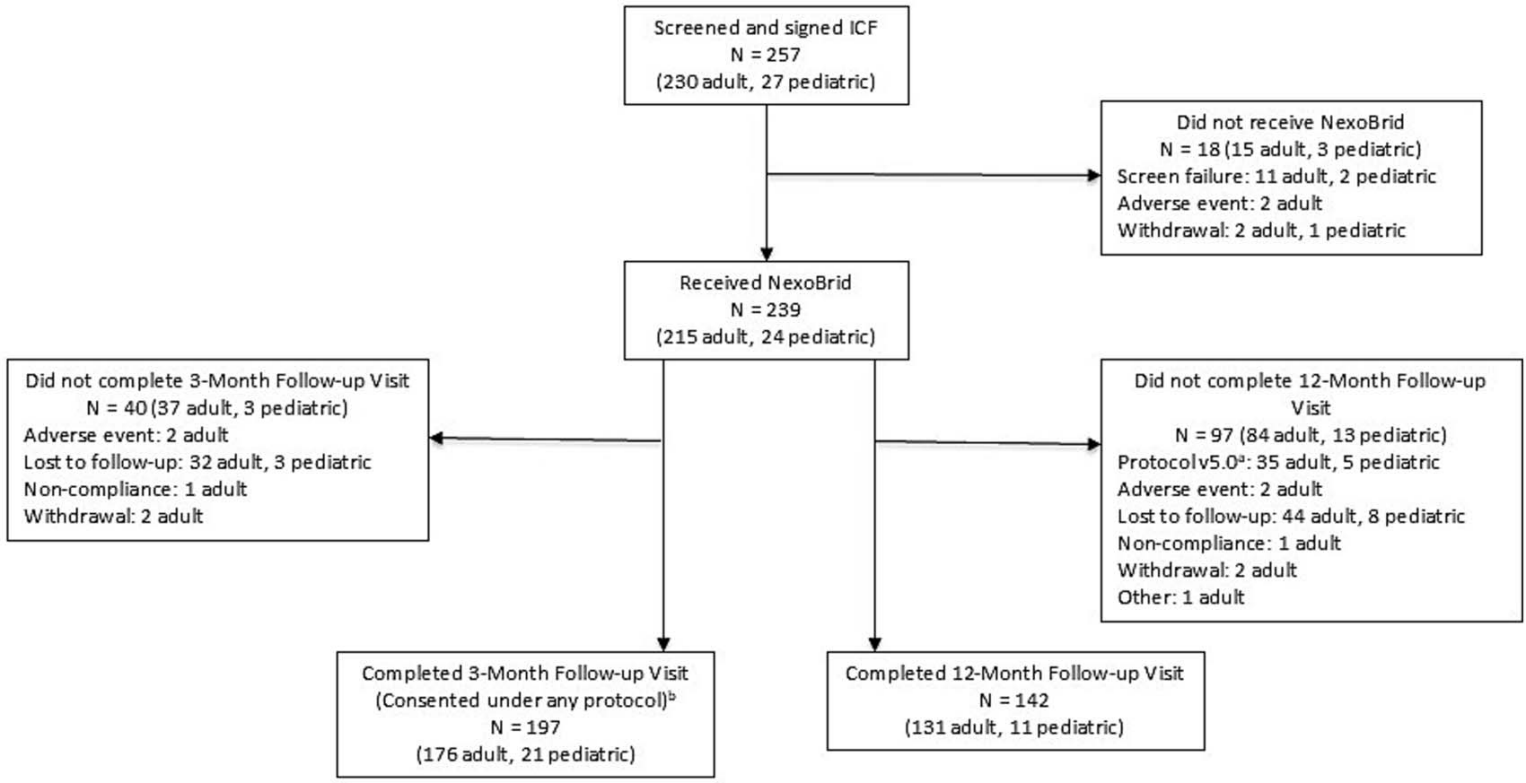
Patient Disposition Flow Chart (Enrolled Population)**.** ICF, Informed Consent Form; TBSA, Total Body Surface Area. (A) Patients Who Enrolled Under Protocol Version 5.0 were not Required to Complete the 12-Month Follow-Up Visit. (B) An Additional Two Adult Patients With TBSA ≤15% did not Complete the 3-Month Follow-Up Visit, but did Complete the 12-Month Follow-Up Visit.

A total of 257 patients were screened and 239 enrolled for this EAP, of whom 215 adult and 24 pediatric patients received treatment with NexoBrid in 23 burn centers (Figure 1). Four centers enrolled more than 20 patients each, with one site enrolling 37 patients (range: 2–37). A total of 197 (82.4%) patients (176 adult, 21 pediatric) who received NexoBrid completed the 3-month follow-up visit. A total of 142 (59.4%) patients (131 adult, 11 pediatric) completed the 12-month follow-up visit. The most common reasons for not completing the 12-month follow-up visit were loss to follow-up (44 adult and 8 pediatric patients) and earlier completion (34 adult and 5 pediatric patients) per protocol version 5, which required follow-up for only 3 months post-wound closure. Two adult patients did not complete the 12-month follow-up visit due to AEs of hypersensitivity possibly related to NexoBrid and ischemic stroke not related to NexoBrid, each in one patient.

Demographic characteristics are presented in Table 1.

**Table 1:** Summary of Demographics and Baseline Characteristics—Patient Level (Safety/ITT Population)

	Adult patients ^a^ (*N* = 215)	Pediatric patients (*N* = 24)
Age in years, mean (SD)	40.7 (15.5)	10.9 (5.6)
Sex (%)		
Female	51 (23.7)	7 (29.1)
Male	164 (76.3)	17 (70.9)
Race (%)		
Asian	2	0
Black or African American	54	7
White	143	16
Native Hawaiian or other Pacific Islander	1	0
Other	15	0
Burn etiology, *n* (%)		
Contact	11 (5.1)	2 (8.3)
Fire or flame	140 (65.1)	13 (54.2)
Scald	64 (29.8)	9 (37.5)
Total burn area (% TBSA), mean (SD)	9.1 (7.4)	9.5 (11.1)
Burn area (by % TBSA categories), *n* (%)		
≤15%	175 (81.4)	21 (87.5)
>15% and ≤30%	37 (17.2)	1 (4.2)
>30%^b^	3 (1.4)	2 (8.3)
Target wound area (%TBSA), mean (SD)	6.5 (5.1)	5.7 (4.4)
Target wound depth (%TBSA), mean (SD)		
Superficial partial thickness	0.6 (1.1)	0.7 (1.1)
Deep partial thickness	4.3 (4.3)	3.8 (3.9)
Full thickness	1.6 (2.8)	1.3 (1.4)
Circumferential burns *n* (%)	34 (15.8)	4 (16.7)

^a^Similar distribution of adult population based on NBR, 2019.

^b^Treated target wounds did not exceed 30% TBSA.

Abbreviations: BMI, body mass index; *N*, number; SD, standard deviation.

The mean age was 40.7 years for adult patients and 10.9 years for pediatric patients. Most patients were male and presented with either flame or scald burns. The majority of patients, both adult (81.4%) and pediatric (87.5%), had a total % TBSA for all wounds of ≤15% (Table 1). The mean TBSA was 9.1% and 9.5% in adult and pediatric patients, respectively, while the mean treated TW area was 6.5% and 5.7% in adult and pediatric patients, respectively. Most of the TW areas were assessed as DPT, followed by FT. Thirty-eight patients (34 adult and 4 pediatric) had circumferential burns.

### Clinical performance results

All pediatric patients and the majority of adult patients (197/215 patients) received only one NexoBrid application. Eighteen (8.4%) adult patients had 2 NexoBrid applications in at least one TW. Per protocol, NexoBrid could not be applied in a single application to a TW area larger than 15% TBSA. In these cases, 2 applications were required per protocol. Ten patients underwent a second application because the TW was larger than 15%. Nine additional adult patients (4.1%) had a second application due to incomplete eschar removal after the first application.

NexoBrid achieved complete eschar removal in the vast majority of treated patients (95% adult and 100% pediatric) (Table 2). The calculated median time to complete eschar removal was 0.17 days (4 hours).

**Table 2:** Eschar Removal and Incidence of Excisions

	Adult patients (*N* = 215)	Pediatric patients (*N* = 24)
Complete ER rate (%)	94.9 (204)	100 (24)
Median time to complete ER^a^ (days) (95% CI)	0.17^b^ (NA, NA)	0.17 (NA, NA)
Proportion of patients requiring excision (95% CI)	4.2% (9) (1.9, 7.8)	0% (0) (NA, NA)
% Area excised, mean (SD)	3.6 (18.3)	0 (0)

^a^In NEXT, “time to” was from start of treatment (first application of NexoBrid).

^b^Median time to complete ER in the 8 patients with target wound area larger than 15% was 1.11 days.

Abbreviations: CI, confidence interval (binomial distribution); ER, eschar removal (complete ER is defined as ≥95% of the eschar removed); *N*, number; NA, could not be calculated due to the distribution of the results across the patients; SD, standard deviation.

The incidence of NexoBrid-treated patients who underwent surgical excision for eschar removal was 4.2% and 0% in adult and pediatric patients, respectively. The mean area excised was 3.6% and 0% TBSA in adult and pediatric patients, respectively.

None of the 38 patients with circumferential extremity TWs who were monitored for clinical signs of BICS received a surgical escharotomy.

The median duration of hospitalization during NEXT was 10 days for both adult and pediatric patients (95% CI, 8-11 for adults and 5-14 days for pediatric patients). For the 8 patients with a TW area larger than 15%, the median duration of hospitalization was 28 days.

### Safety results

Kaplan-Meier estimates for the median time to complete wound closure at the TW level were 22.0 days (*n* = 426; 95% CI, 22.0-23.0) for adult patients and 28.0 days (*n* = 35; 95% CI,18.0-32.0) for pediatric patients (Table 2). Results were similar in the patient-level analysis, with 23.0 days (*n* = 215; 95% CI, 22.0-25.0) for adult patients and 27.0 days (*n* = 24; 95% CI, 16.0-32.0) for pediatric patients.

**Figure 2:**
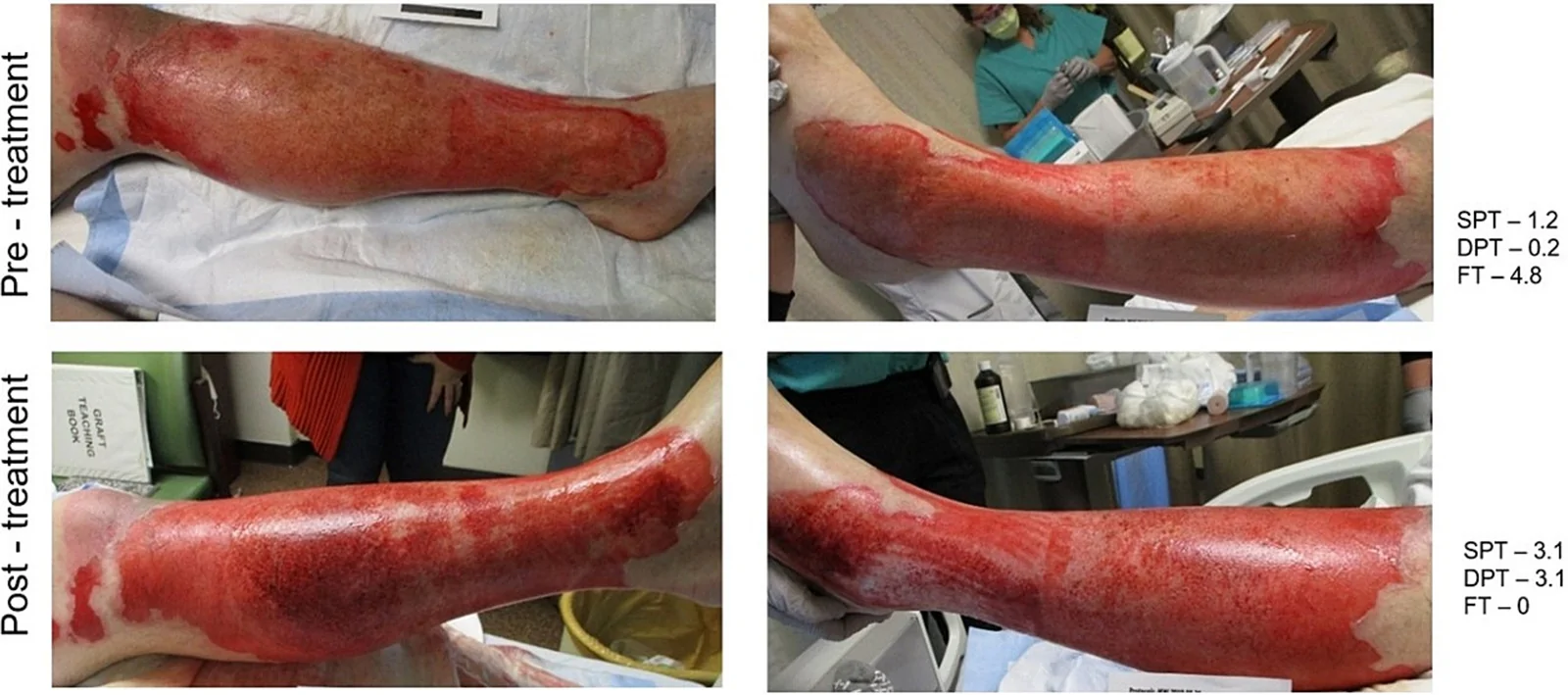
Case Example of a 49-Year-Old Male With a Flame Burn Injury, Treated With NexoBrid® In The NEXT Expanded Access Program. Left Lower Leg Flame Burn in a 49-Year-Old Male Treated With NexoBrid® In NEXT. Upper Left: Medial Aspect Before Treatment. Lower Left: Medial Aspect After Treatment. Upper Right: Lateral Aspect Before Treatment. Lower Right: Lateral Aspect After Treatment. Burn Depth (%TBSA) Classified as Superficial Partial Thickness (SPT), Deep Partial Thickness (DPT), and Full Thickness (FT), as Assessed Pre- and Posttreatment. All images courtesy of Dr. Lucy A. Wibbenmeyer, MD, FACS, Department of Surgery, University of Iowa Health Care.

**Figure 3:**
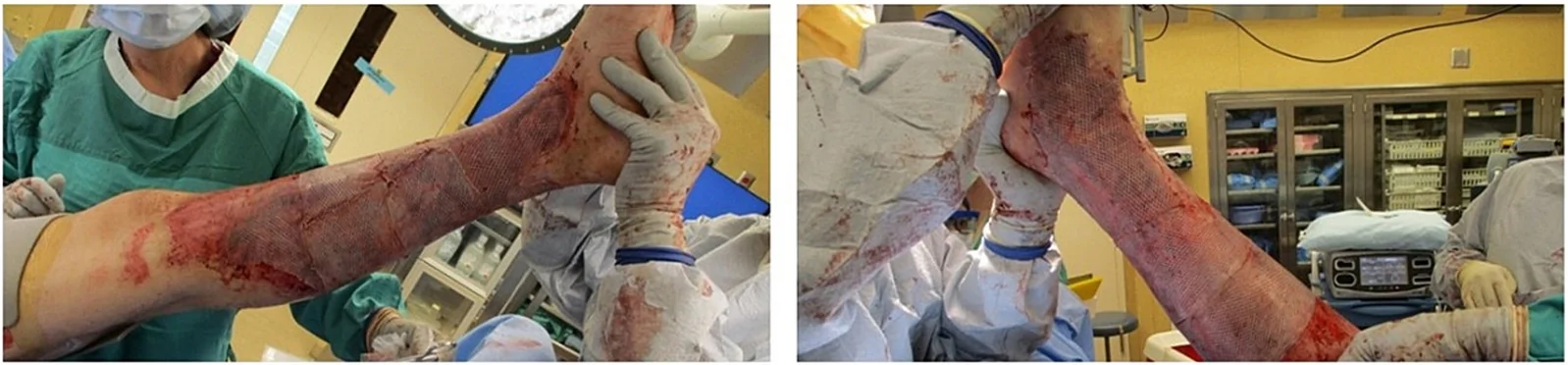
Same Patient Shown in Figure 2. Wound Coverage With Autograft 8 Days After NexoBrid® Treatment. *Left*: Medial Aspect. *Right*: Lateral aspect.

**Figure 4:**
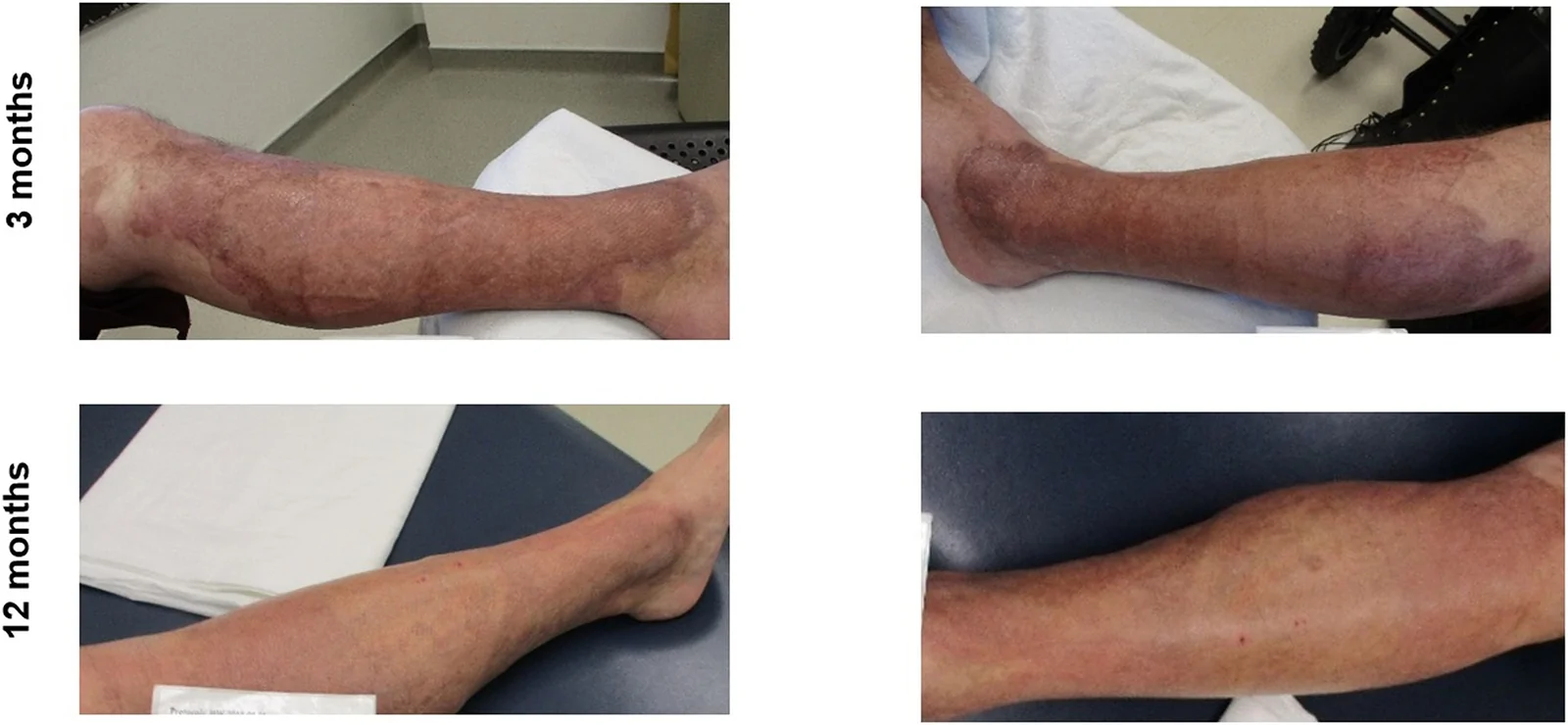
Same Patient as in Figures 2 and 3. Scar Outcomes at 3 Months and 12 Months Following Wound Closure. *Left*: Medial Aspect. *Right*: Lateral Aspect.

Cosmesis and function assessment were performed based on the MVSS at 12 months after the wound closure confirmation visit. MVSS scores were similar in the adult and pediatric populations (mean scores 4.5 [±3.3] and 5.6 [±3.9], respectively). Figures 2–4 present a case study of an adult patient treated with NexoBrid, illustrating the long-term scar outcome.

Overall, treatment with NexoBrid was safe and well-tolerated. No clinically meaningful safety differences were observed between adults and children.

Treatment-Emergent Adverse Events (TEAEs) were mild or moderate for the majority of patients. Wound infection was the most common serious TEAE (Table 3). Most TEAEs were considered not related to NexoBrid by the investigator.

**Table 3:** Occurrence of Treatment-Emergent Adverse Events

Event summary		NEXT
Adult subjects (*N* = 215) *n* (%)	All AEs	123 (57.2)
	SAEs	11 (5.1)
	Severe	8 (3.7)
	Related AEs	22 (10.2)
	Death	1 (0.5)
Pediatric subjects (*N* = 24) *n* (%)	All AEs	13 (54.2)
	SAEs	6 (25.0)
	Severe	2 (8.3)
	Related AEs	3 (12.5)
	Death	0

Abbreviations: AEs, adverse events; *N*, number; SAEs, serious adverse events.

The most commonly reported TEAEs (Table 4) were wound infection, pyrexia, and pruritus among adult and pediatric patients. In pediatric patients, vomiting and coronavirus infections were also commonly reported.

**Table 4:** Summary of Treatment-Emergent Adverse Events, by Preferred Term, in >2% (4) Adult Patients or >8% (2) Pediatric Patients, Anytime During the Program

Adverse event by preferred term *n* (%)	Adult patients (*N* = 215)	Pediatric patients (*N* = 24)
Wound infection	21 (9.8%)	4 (16.7%)
Pyrexia	21 (9.8%)	2 (8.3%)
Pruritus	15 (7.0%)	3 (12.5%)
Cellulitis	10 (4.7%)	0
Hypertension	9 (4.2%)	1 (4.2%)^a^
Pain	9 (4.2%)	1 (4.2%)^a^
Vomiting	6 (2.8%)	2 (8.3%)
Anemia	6 (2.8%)	0
Graft loss	6 (2.8%)	0
Constipation	5 (2.3%)	1 (4.2%)^a^
Wound complication	5 (2.3%)	1 (4.2%)^a^
Insomnia	5 (2.3%)	0
Tachycardia	5 (2.3%)	0
Coronavirus infection	2 (1.0%)	2 (8.3%)

^a^One patient.

Wound infection (4 [1.9%] adult patients, 2 [8.3%] pediatric patients) was the most common serious TEAE. One serious TEAE of wound infection and one serious TEAE of systemic inflammatory response syndrome were considered to be possibly related or probably related to NexoBrid, respectively, as assessed by the study investigator; all other serious TEAEs were considered not related to NexoBrid.

No clinically concerning results pertaining to hematology, biochemistry, coagulation, or vital signs were observed.

Among adult patients, the mean NPRS score was 4.2 at baseline and 4.5 posttreatment, demonstrating no clinically meaningful change from baseline in the patient’s perception of pain. Among pediatric patients, the mean FPS-R score was 1.4 at baseline and 0.7 posttreatment, demonstrating no clinically meaningful change from baseline in the patient’s perception of pain.

## DISCUSSION

The NEXT EAP was initiated to allow treatment with NexoBrid in the United States for eschar removal in adult and pediatric burn patients, once enrollment was complete in either the DETECT or CIDS clinical trials. This EAP was designed to collect and evaluate real-world data on the safety and clinical performance of NexoBrid and maintain burn care preparedness for burn mass casualty incidents in the United States. Specifically, maintaining clinical experience with NexoBrid, including product preparation, application, and post-application wound management, may help preserve institutional familiarity and operational readiness in participating burn centers in the event of a surge of burn patients.

The NEXT EAP was the largest medical interventional protocol for eschar removal performed to date in burn care. The patient population treated with NexoBrid in the NEXT EAP was larger than the pooled population of the DETECT and CIDS trials, and the results obtained in this large population constitute strong confirmation of the safety and efficacy of NexoBrid demonstrated in previous clinical trials.

The NEXT program was conducted as an open-label, single-arm expanded access initiative. Any comparisons discussed herein with previously published Phase III trials are cross-study in nature and should be interpreted cautiously given differences in study design, patient populations, and endpoints.

This EAP was conducted exclusively in the United States as an extension of the DETECT and CIDS trials. As a result, many participating clinical sites had prior experience with NexoBrid through these earlier studies. The increased patient enrollment observed in NEXT compared to previous trials may reflect growing confidence among healthcare providers in the use of NexoBrid, as well as broader familiarity with the product gained during the prior trials.

The %TBSA of TWs per patient treated with NexoBrid was comparable across the NEXT, DETECT, and CIDS studies. In NEXT, the mean %TBSA of TWs was 6.5% in adults (*n* = 215) and 5.7% in pediatric patients (*n* = 24). In DETECT (*n* = 75) and CIDS (*n* = 72), mean %TBSA was 6.28% and 5.85%, respectively. Wound depth distribution was also consistent: In NEXT, DPT and FT wounds accounted for 4.3% and 1.6% TBSA in adults, and 3.8% and 1.3% in pediatric patients. In DETECT and CIDS, DPT/FT wounds represented 3.8%/1.6% and 4.0%/0.7% TBSA, respectively.

The NEXT EAP demonstrated that NexoBrid achieved rapid and complete eschar removal without the need for surgery in the vast majority of treated patients (95% adult and 100% pediatric), consistent with the outcomes reported in the DETECT (93%) and CIDS (90%) trials.

Eschar removal in the NEXT EAP was rapid, with a median time of approximately 0.17 days (4 hours), compared to 1.02 and 0.99 days in the DETECT and CIDS trials, respectively, for patients treated with NexoBrid, and 3.83 and 5.99 days for those receiving standard of care. The shorter duration observed in NEXT is primarily due to differences in the assessment method: In NEXT, time to eschar removal was calculated from treatment initiation, while in DETECT and CIDS, it was measured from randomization. These data demonstrate that eschar removal with NexoBrid was consistently rapid across both clinical trials and the EAP.

Data from the NEXT EAP indicated that the need for surgical eschar excision was minimal, with only 4% of adult patients and none of the pediatric patients requiring the procedure. The minimal number of patients requiring surgical eschar excision may be attributed to incomplete debridement resulting from specific eschar characteristics (eg, hard to remove keratin layers), or suboptimal application techniques. These findings are consistent with those observed in the NexoBrid arms of the DETECT and CIDS trials, where surgical excision was performed in 4% and 8.3% of patients, respectively. For contextual reference, in the standard-of-care control arms of the DETECT and CIDS Phase III trials, 72% and 64.4% of patients, respectively, received surgical excision.

The NEXT EAP included circumferential extremity TWs. Of 34 adult and 4 pediatric patients monitored for BICS, no escharotomies were performed. These findings should be interpreted with caution as the study did not include a comparator arm and the number of circumferential burns was relatively limited. Importantly, the absence of escharotomies in this cohort does not establish prevention of compartment syndrome. Rather, these real-world observations may be considered hypothesis-generating and are consistent with prior reports suggesting that early enzymatic eschar removal may mitigate progressive pressure buildup in circumferential burns and potentially reduce the need for surgical escharotomy.^8^^,^^15^ Prospective controlled studies with standardized monitoring are required to formally evaluate this potential benefit.

In the NEXT EAP, the median time to wound closure was 22 and 28 days in adult and pediatric patients, respectively. These outcomes are in line with the NexoBrid arms of previous controlled trials, with median closure times of 27 days in adult patients (DETECT) and 32 days in pediatric patients (CIDS) (median time to wound closure in the standard of care arm in the DETECT and CIDS trials was 28 days and 34 days, respectively). The time to wound closure observed in NEXT is consistent with previously reported data from US centers and further supports the reproducibility and effectiveness of NexoBrid in clinical practice.

The median duration of hospitalization in the NEXT EAP was 10 days for both adult and pediatric patients. This was numerically consistent with the durations observed in the NexoBrid arms of the DETECT and CIDS trials (14 and 12 days, respectively), and within the range reported in the standard of care arms of the same studies (12 and 10 days, respectively). These findings are also consistent with previously reported data from US centers.

Long-term mean scar outcome scores, as assessed by the MVSS at 12 months, in the NEXT EAP, were 4.5 in adults and 5.6 in pediatric patients. With the caveat of cross trials comparison, these scores were numerically higher (indicating worse outcomes) than those reported in the NexoBrid arms of the DETECT and CIDS trials (both 3.7), but comparable to those in the standard of care arms (5.1 and 4.65, respectively). Scar outcome (MVSS) reflects wound severity (baseline wound characteristics) and treatment pathway. The baseline characteristics of TWs treated with NexoBrid with MVSS assessment at 12 months were comparable across the NEXT (*N* = 264), DETECT (*n* = 81), and CIDS (*n* = 77) studies. Mean %TBSA of TWs was 3.2% (SD 2.7) in NEXT, 3.9% (SD 2.4) in DETECT, and 4.4% (SD 3.1) in CIDS. The DPT component averaged 2.1% (SD 2.2), 2.4% (SD 1.5), and 2.8% (SD 2.5) in NEXT, DETECT, and CIDS, respectively, while the FT component was 0.8% (SD 1.4), 1.0% (SD 1.9), and 0.7% (SD 1.9), respectively.

In the NEXT EAP, MVSS scores were higher for autografted wounds compared to non-autografted wounds (5.76 vs 3.24, respectively), a finding that reflects the deeper nature of wounds selected for autografting (mean %TBSA of FT TWs was 1.0% [SD 1.5] in autografted wounds vs. 0.6% [SD 1.1] in non-autografted wounds). Notably, 48.9% of DPT wounds in NEXT were treated with autografting, nearly double the proportion observed in DETECT (25.4%) and CIDS (26.1%). In the absence of noted differences in wound severity across NEXT, DETECT, and CIDS, the numerically higher MVSS scores observed in NEXT may be associated with the higher rate of autografting for DPT wounds in the NEXT EAP. However, other differences in baseline characteristics or treatment pathways that were not captured or formally assessed may also have contributed to these results.

The safety of NexoBrid, as evaluated in the NEXT EAP through analyses of TEAEs, was favorable and consistent with the known safety profile of NexoBrid from prior clinical trials (DETECT and CIDS) and the numerous publications and consensus papers^11^^,^^16^ published to date on the commercial use of NexoBrid.

The overall frequencies of TEAEs reported during the NEXT EAP were consistent with those reported in the controlled clinical trials (DETECT and CIDS). In both Phase III trials, the overall frequencies of TEAEs were similar between the NexoBrid and control arms. In adult NEXT patients, the overall frequency of TEAEs was 57.2%, similar to the DETECT trial (63.6% with NexoBrid, 60.3% in the control). In pediatric NEXT patients, the overall frequency of TEAEs was 54.2%, similar to the CIDS trial (46.4% with NexoBrid, 44.3% in the control). Similar to the CIDS and DETECT trials, the vast majority of TEAEs in the NEXT EAP were assessed by the investigators as mild or moderate in severity. Most TEAEs were considered not related to NexoBrid by the investigator in the NEXT EAP as well as in the NexoBrid arms of the controlled trials (10.2% vs 11.7% in the NEXT and DETECT trials, respectively, with 12.5% vs 13% of the TEAEs in the NEXT and CIDS trials, respectively, reported as possibly related to NexoBrid). Wound infection, pyrexia, and pruritus were the most common TEAEs in NEXT. In previous trials, wound infection and pyrexia were comparable to standard of care.

## CONCLUSION

The NEXT EAP successfully preserved physician expertise, ensured continued access to NexoBrid for burn patients, and enabled the collection of real-world efficacy and safety data, while also enhancing national preparedness for burn mass casualty incidents.

A key distinguishing feature of the program was its exclusive implementation in the United States, at many centers with prior experience using NexoBrid in earlier trials such as DETECT and CIDS. This prior familiarity likely contributed to robust patient enrollment and favorable clinical outcomes. Efficacy measures, including rates of complete eschar removal, time to wound closure, and length of hospital stay, were consistent with those reported in earlier controlled trials. These findings underscore the importance of robust training and hands-on experience for new centers to achieve similar outcomes.

Notably, the NEXT EAP also provided supportive evidence of the potential of NexoBrid to prevent compartment syndrome in circumferential burns, while maintaining a favorable and consistent safety profile.

Collectively, these findings reinforce the reproducibility of NexoBrid’s favorable benefit-risk profile and underscore its role as a valuable non-surgical option for effective eschar removal in contemporary burn care.

## Role of Funder

BARDA had no role in the design or conduct of the study. MediWound Ltd. provided the treatment agents.

## Notes

Author contributions: Steven A. Kahn (Investigation [equal], Writing—original draft [equal], Writing—review & editing [equal]), John Schulz (Investigation [equal], Writing—original draft [equal], Writing—review & editing [equal]), Tam Pham (Investigation [equal], Writing—original draft [equal], Writing—review & editing [equal]), Andrew Clinton Bright (Investigation [equal], Writing—original draft [equal], Writing—review & editing [equal]), Dhaval Bhavsar (Investigation [equal], Writing—original draft [equal], Writing—review & editing [equal]), Nicole Bernal (Investigation [equal], Writing—original draft [equal], Writing—review & editing [equal]), Steve Sandoval (Investigation [equal], Writing—original draft [equal], Writing—review & editing [equal]), and Robert Bertellotti (Investigation [equal], Writing—original draft [equal], Writing—review & editing [equal])

Funding: This project has been supported in whole or in part with federal funds from the Department of Health and Human Services; Administration for Strategic Preparedness and Response; Biomedical Advanced Research and Development Authority, under Contract No. HHSO100201500035C. The findings and conclusions in this publication have not been formally disseminated by the Department of Health and Human Services and should not be construed to represent any agency determination or policy.
